# AT_CHLORO: A Chloroplast Protein Database Dedicated to Sub-Plastidial Localization

**DOI:** 10.3389/fpls.2012.00205

**Published:** 2012-09-11

**Authors:** Christophe Bruley, Véronique Dupierris, Daniel Salvi, Norbert Rolland, Myriam Ferro

**Affiliations:** ^1^CEA, DSV, IRTSV, Laboratoire Biologie à Grande Echelle, Institut de Recherches en Technologie et Sciences pour le VivantGrenoble, France; ^2^INSERM, U1038Grenoble, France; ^3^Université Joseph Fourier, Grenoble 1Grenoble, France; ^4^CEA, DSV, IRTSV, Laboratoire de Physiologie Cellulaire et VégétaleGrenoble, France; ^5^CNRS, UMR5168Grenoble, France; ^6^INRA, USC1359Grenoble, France

**Keywords:** chloroplast, proteomics, sub-plastidial localization, chloroplast envelope, database

## Abstract

AT_CHLORO (www.grenoble.prabi.fr/at_chloro) is a database dedicated to sub-plastidial localization of *A. thaliana* chloroplast proteins. This information was infered from proteomics experiments obtained from a comprehensive study that allowed the identification of proteins from envelope, stroma, and thylakoid sub-compartments Ferro et al., 2010. In addition to current knowledge regarding sub-plastidial localization, AT_CHLORO provides experimental data that allowed curated information regarding subcellular localizations of chloroplast proteins to be given. A specific focus was given to proteins that were identified in envelope fractions and for which expert functional annotation was provided. The present mini review shows the specificities of AT_CHLORO with respect to available information, data export options and recent improvements in data representation.

## Introduction

As chloroplasts are Earth’s main solar energy converters, much interest has emerged for a better knowledge of chloroplast biology. Indeed, most renewable carbon is fixed by photosynthetic organisms through a process made possible by their chloroplasts. Being the location of essential metabolic pathways (photosynthesis, synthesis of lipids, pigments, amino acids, vitamins, starch, precursors of plant hormones, etc.), the chloroplast is an established target for metabolic engineering of crop plants to improve productivity. In order to carry out such an array of biochemical reactions, chloroplasts need to coordinate the functions of three main sub-compartments: the envelope, the stroma, and the thylakoids. The envelope comprises a pair of membranes surrounding the chloroplast and controls the dialog between the chloroplast and the rest of the cell (Block et al., 2007). In addition, those membranes are also involved in many other essential metabolic reactions (e.g., lipid, pigment, or vitamin synthesis). The stroma, the soluble phase of the chloroplast, is the main place for the conversion of carbon dioxide into carbohydrates. Other catalytic reactions occur in the stroma that allow the synthesis of compounds such as amino acids. The thylakoids are a highly organized internal membrane network where solar energy is collected and converted into chemical energy (ATP and NADPH).

In order to investigate chloroplast metabolism and main functions, sub-plastidial localization is a crucial piece of information required to select proteins in the context of targeted functional characterization. In that context, recent advances in the proteomic field have allowed high throughput experiments to be conducted on chloroplast samples and to provide additional information about functional compartmentalization (Agrawal et al., 2011; van Wijk and Baginsky, 2011). The spatial distribution of proteins within chloroplasts has been investigated from various independent studies which aimed to establish the proteome repertoire of sub-plastidial compartments: the thylakoids (e.g., Friso et al., 2004; Peltier et al., 2004), the stroma (e.g., Zybailov et al., 2008), the plastoglobules (e.g., Vidi et al., 2006; Ytterberg et al., 2006; Lundquist et al., 2012), and the envelope (e.g., Ferro et al., 2002, 2003; Froehlich et al., 2003). This targeted repertoire allowed the identification of minor components of each of these sub-compartments. Whereas these repertoires are highly informative, the actual sub-plastidial localization of some proteins might be questionable as they were identified in different chloroplast sub-fractions or in other subcellular compartments. Indeed, the actual localization of proteins within a fraction that has been used for proteomics analyses is related to cross-contamination issues. Thus, MS-based quantification strategies, must be applied to discriminate between true and false protein localization assignments (e.g., Dunkley et al., 2006). In that context, as the accurate localization of many chloroplast proteins remained hypothetical, we set up a proteomics strategy which aimed to ascertain the sub-plastidial localization of chloroplast proteins (Ferro et al., 2010). Using a MS-based semi-quantitative strategy (spectral count), we chose to revisit the sub-plastidial localization of chloroplast proteins. In order to gage sub-plastidial cross-contamination, we started from purified sub-fractions retrieved from the same chloroplast samples. MS-based sub-plastidial localization of identified proteins was stored in the AT_CHLORO database^1^ which compiles results from LC-MS/MS analyses of highly purified sub-fractions of the three major chloroplast sub-compartments. From the MS analyses, about 1,300 proteins were identified, of which more than 800 proteins could be assigned a sub-plastidial localization. In addition, the AT_CHLORO database condenses public and curated information related to protein function and localization, especially for envelope proteins.

## Presentation of the Database

**Figure 1:**
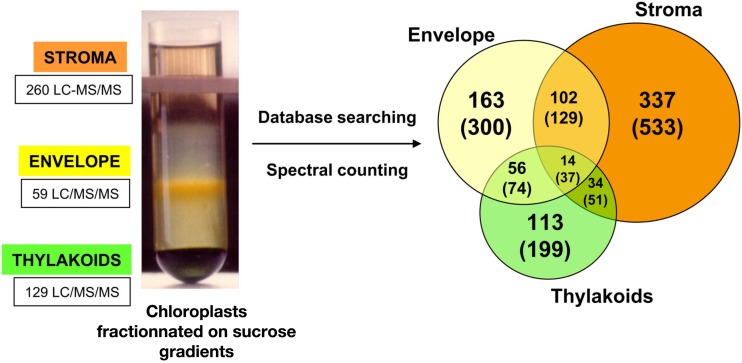
**Distribution of protein sub-plastidial localizations as determined using spectral count data for a subset of 819 selected proteins**. Number into brackets correspond to the total 1323 proteins. Left part: fractionation of chloroplasts on a sucrose gradient (0.3, 0.6, and 0.93 M sucrose layers from top to bottom) after osmotic shock performed on Percoll-purified chloroplasts. After centrifugation, the envelope membrane and the thylakoids are present as a yellow band at the 0.93/0.6 M interface and as a dark green band at the bottom of the tube, respectively. The soluble fraction containing the stroma remains on top of this gradient (see, Salvi et al., 2011).

AT_CHLORO is one of the databases dedicated to the chloroplast proteome of *Arabidopsis thaliana* and specifically gathers information for proteins that have been identified from the main chloroplasts sub-fractions: envelope, stroma, and thylakoids (Demartini et al., 2011). Sub-plastidial localization was assessed using MS-based data corresponding to the three purified chloroplast compartments – envelope, thylakoids, and stroma – that had been, for the first time, analyzed in the same set of experiments (Ferro et al., 2010). Briefly, purification of those three chloroplast sub-fractions was achieved using sucrose gradients (Salvi et al., 2011). Envelope, stroma, and thylakoid fractions were either digested in solution or analyzed by SDS-PAGE prior to trypsin digestion (Salvi et al., 2008). Then, generated samples were submitted to LC-MS/MS analysis for identification purposes. About 500 LC-MS/MS analyses were performed, ending up with the identification of 1,323 proteins. As chloroplast sub-fractions were prepared with a low level of contamination, as determined by Western blot analyses, and starting from the same chloroplast samples, semi-quantitative spectral count data allowed assessment of protein relative abundances in each of the three sub-fractions. Thus, the partitioning of each of the 1,323 proteins in envelope, stroma, and thylakoids was calculated based on normalized spectral count data, from which a percentage of occurrence in each sub-compartment was deduced. Amongst the 1,323 proteins, statistical analysis allowed the accurate localization of 819 proteins (Figure 1).

Four types of information can be found in the AT_CHLORO database: (i) the proteomics-based sub-plastidial localization as revealed by spectral counting; (ii) analytical coordinates (HPLC retention time, RT; peptide molecular weight, Mr) of all the peptides corresponding to the proteins stored in the database; (iii) curated localization and function of proteins, especially the ones that were identified in envelope fractions, and (iv) information from public databases such as TAIR^2^, or PPDB^3^ (Sun et al., 2009). Some data from AT_CHLORO can also be retrieved from the MASCGator Portal (Joshi et al., 2011). Information related to the sub-plastidial localization was also submitted to TAIR.

## Description of the Different Types of Information

AT_CHLORO gathers three levels of information: MS-based experimental data, curated annotations, and public information. All this information gives a comprehensive overview of current knowledge about the localization and the function of identified chloroplast proteins. Definitions of the different types of information can be found in Table 1. Fields or columns in which the different types of information can be found appear in italics in the text below.

**Table 1:** **Description of the features found in AT_CHLORO**.

Experimental data; subplastidial localizations (see Ferro et al., 2010)	Description
ENV (SC)	Envelope spectral count: % of spectra recovered in the purified envelope fraction
STR (SC)	Stroma spectral count: % of spectra recovered in the purified stroma fraction
THY (SC)	Thylakoid spectral count: % of spectra recovered in the purified thylakoid fraction
Total (SC)	Total spectral count retrieved from the AT_CHLORO database
Localization (SC): *definition of the values*
*ENV*	Localization in the envelope
*STR*	Localization in the stroma
*THY*	Localization in the thylakoids
*ENV-STR*	Dual localization in the envelope and the stroma, preferentially in the envelope
*ENV-THY*	Dual localization in the envelope and the thylakoids, preferentially in the envelope
*STR-ENV*	Dual localization in the stroma and the envelope, preferentially in the stroma
*STR-THY*	Dual localization in the stroma and the thylakoids, preferentially in the stroma
*THY-ENV*	Dual localization in the thylakoids and the envelope, preferentially in the thylakoids
*THY-STR*	Dual localization in the thylakoids and the stroma, preferentially in the thylakoids
*Mix*	No obvious sub-plastidial localization
-	No sub-plastidial localization (spectral count < 10)

**Experimental data; analytical information (see Ferro et al., 2010)**	**Description**

Sequence	Peptide amino acid sequence
Monoisotopic mass	Theoretical peptide monoisotopic mass
peptides retention time	Normalized peptide retention time on a C18 LC column
score	The highest Mascot score that allowed peptide assignment
Observed MS/MS count	Observed MS/MS count
Curated annotations (see Ferro et al., 2010)
Localization (curated)	Subcellular or sub-plastidial localization compiled from Ferro et al. (2010), previous proteomic studies, targeting prediction and bibliography.
Function (curated)	Manual annotation of the functional classification
References	Literature references, crude data extracted from various protein databases,% of similarity with orthologs or paralogs, domains etc.
Description (curated)	Curated description
Localization (validated)	Localization validated by additional experiments (e.g., GFP fusions) as described in the literature
Localization (curated): *definition of the values*
*Ch*	Chloroplast
*E*	Envelope
*IM*	Inner membrane
*OM*	Outer membrane
*other*	Non-plastid localization
*S*	Stroma
*Th*	Thylakoids
*?*	Putative localization
**INFORMATION FROM PUBLIC DATABASES**	
Accession	AGI accession number; gene name
Master Protein	AGI protein accession number. This protein is the representative of a protein group that shares the same set or a subset of peptides.
Description (TAIR)	Description (Standard Annotation) retrieved from TAIR
Length	number of amino acids
Calculated PI (PPDB)	Calculated isoelectric point. Information retrieved from PPDB.
Calculated MW (PPDB)	Molecular mass of the protein in kiloDalton. Information retrieved from PPDB.
Localization (TAIR)	Subcellular or sub-plastidial localization as described in TAIR.
MapManBin (PPDB)	MapMan functional classification. Information retrieved from PPDB.
ChloroP	Prediction of plastid localization using ChloroP (http://www.cbs.dtu.dk/services/ChloroP/)
Curated localization (PPDB)	Curated subcellular or sub-plastidial localization as stated in PPDB.
TargetP	Prediction of subcellular localization using TargetP (http://www.cbs.dtu.dk/services/TargetP/)
Aramemnon	Number of transmembrane helices as found in the Aramemnon database (http://aramemnon.botanik.uni-koeln.de/)
Publications (PPDB)	List of most subcellular targeted proteomics studies (MedLine numbers) in which the protein was identified. Information retrieved from PPDB.
ChloroP: *definition of values*
*Y*	The protein has a predicted chloroplast transit peptide
TargetP: *definition of values*
*C*	Chloroplast
*M*	Mitochondria
*SP*	Secretory pathway
*–*	No prediction

*“?” indicates that the proposed subcellular or subplastidial localizations remain putative. In the database, the “?” is associated to localization classes (e.g. Ch?, Ch/E?, …)*.

### Experimental data: Proteomics-based sub-plastidial localization as deduced from spectral counting

Experimental data were extracted from (Ferro et al., 2010). Briefly, for each protein and each chloroplast sub-fraction (envelope, stroma, and thylakoids), the number of associated spectra was retrieved from LC-MS/MS and database searching data. Because spectral counting is a semi-quantitative approach, significant ratios, and thresholds are generally high. Consequently, only 819 proteins identified with at least 10 spectral counts were taken into account for being assigned an accurate sub-plastidial localization. Spectral counts were normalized with respect to the number of assigned MS/MS spectra in each fraction and a percentage of occurrences in each sub-fraction was calculated for all proteins (*ENV SC; STR SC; THY SC*). The localization given by normalized spectral counts was verified using a logistic regression model. From the calculated percentages, proteins were attributed a single, dual, or mixed sub-plastidial localization. A single localization was thus assigned to proteins for which the percentages of occurrence in the two other sub-fractions were below a threshold level fixed at 15%. This percentage was set to 15%, above the cross-contamination level as estimated by Western blotting. Dual localization was assigned to proteins with a major localization (occurrence ≥50%) and a secondary localization (occurrence ≥15%). The remaining proteins were considered to have a mixed localization between the three sub-plastidial compartments [*Localization* (*SC*)]. For all proteins, the total number of spectral counts can also be viewed and gives an assessment of the relative amount of a given protein in the chloroplast [*Total* (*SC*)].

### Experimental data: Analytical coordinates for label-free quantification

The AT_CHLORO database is not only a repository of chloroplast proteins but also gathers information related to peptides that have allowed protein identification. Thus peptide sequences (*sequence*), theoretical molecular weight (*monoisotopic mass*), chromatographic retention times (*Peptides retention time*), the score that allowed peptide identification (*score*), and spectral count (*observed MS/MS count*) can be found in the window dedicated to each protein. Theoretical molecular weight and chromatographic retention times can be particularly useful for label-free based quantification studies using the AMT strategy (Lipton et al., 2006). Indeed, the accurate mass and time tags (AMT) method, combines identification, and quantification issues in the context of high throughput quantitative experiments. In a first stage, standard shotgun proteomics approaches are undertaken on extensively fractionated proteins to yield peptide identification. Those experiments yield a database containing the calculated masses based on putative peptide sequences and their corresponding measured chromatographic retention times. Thus, AT_CHLORO is also an AMT database dedicated to the chloroplast. Accurate mass and time tags can subsequently be used, in the course of “simple” LC-MS measurements, as biomarkers of the presence of a given protein without resorting systematically to MS/MS for identification. Consequently, it becomes possible to identify hundreds of proteins in a single MS spectrum in all subsequent LC-MS experiments, using high resolution mass spectrometers, such as the Orbitrap.

### Curated localization and function of proteins

From experimental data and information retrieved from public repositories, curated localizations and functions were given. As sub-plastidial localization is the main focus of AT_CHLORO special care was taken with regard to localization annotations. Thus experimental sub-plastidial localization, previous proteomic studies, targeting prediction, and bibliography were compiled in order to assign a curated localization [*Localization* (*curated*)]. In this context, since the first release of AT_CHLORO, we improved the curated localization of some proteins by providing information about the lumenal localization of a given set of thylakoid proteins, as selected from two reference papers in the field (Peltier et al., 2002; Schubert et al., 2002). Also manual annotation of protein function was undertaken [*Function* (*curated*)]. A specific emphasis was given to about 700 proteins identified in envelope sub-fractions. Indeed, as most available chloroplast proteomics data provide information about proteomes from thylakoids and stroma compared to the envelope, we paid specific attention to analyzing the proteome of the two envelope membranes. External sources that were used for curated annotations, such as literature references, similarity values with orthologs or protein domains can be found in the *references* field. This recent update of the AT_CHLORO database also includes citation of more recent publications, in the *references* field, for some selected proteins.

### Information from public databases

Public information was retrieved from TAIR^4^ and PPDB^5^ (Sun et al., 2009) and are listed in Table 1.

## How to Use the AT_CHLORO Database

AT_CHLORO is organized around four main types of pages: the main page, the search page, the protein list page and the protein ID page.

### The main page

The main page of AT_CHLORO presents a short description of the data that were generated to build the database with associated references and contact. The top main menu contains six options. The user can choose to get the list of all the proteins identified by (Ferro et al., 2010; *all*) or to visualize the list of proteins specifically identified in one of the three chloroplast sub-fractions (*Envelope, Stroma, Thylakoids*). Also, users can search for a given protein or a list of proteins using different features (*Search*). Results can be viewed by clicking on the “*Search Results”* option. The list of features with associated description can be accessed from the main page.

### The search page

**Figure 2:**
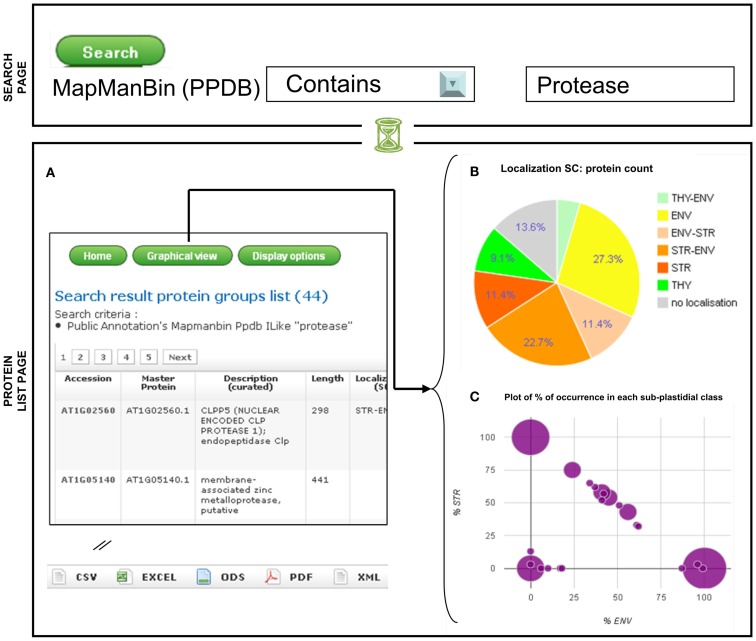
**Visualization of the sub-plastidial localization of a selected set of proteins: example of a search**. Search results **(A)** Protein groups list; **(B)** Pie chart with sub-plastidial localization; **(C)** Plot of% of occurrence in each sub-plastidial class.

The search page can be accessed from the main page top menu using the “*Search*” option. In the first version of AT_CHLORO, proteins could be selected by loading the accession number, or the protein’s description, localization, or function. In the current version it is also now possible to retrieve proteins according to selected values related to one or several features. For instance, proteins can be selected according to a particular function either from the *Function* (*curated*) or the *MapManBin* (*PPDB*) features (Figure 2). As written in the search page, the different search criteria are combined using the AND Boolean Operator.

### The protein list page

A table summarizing the features of a list of proteins can be obtained directly from the main page with the “*All*,” “*Envelope*,” “*Stroma*,” “*Thylakoids*,” “*Search Results*,” options (Figure 2A). Users can customize the display, using the “*Display option*,” button, so that only selected features, amongst the one listed above, are visible on the screen. Recently, graphical representation of the data were added (“*graphical view*” option). As the specificity of AT_CHLORO relies on localization information, proteins can be classified according to their sub-plastidial localization, as given by spectral count data. As shown in Figure 2B, pie-charts can be generated and allow a quick overview of the distribution of proteins within the chloroplast sub-compartments according to the “*Localization (SC)”* feature. A pie chart is available which takes into account the protein counts of “*Localization (SC)”* classes (Figure 2B). Together with the pie-charts, a bar-chart allows a potential enrichment in one of the sub-fraction with respect to the whole database to be assessed. In addition, a diagram which plots the% of occurrence in the envelope according to the% of occurrence in the stroma has been set up which gives a quick overview of the sub-plastidial partitioning of selected proteins (Figure 2C).

From the protein list page it is possible to export data in different formats: csv (comma separated values), xls (Excel), ods (Open Document Spreadsheet), pdf (portable document format), and xml (extensible markup language). Thus the user can easily retrieve protein lists with associated sub-plastidial information for data mining purposes. For instance in a recent report (Tanz et al., 2012) attributes on protein localization were retrieved from AT_CHLORO and were integrated in the widely used Cytoscape tool^6^.

### The protein ID page

From a protein list page, users can select one particular protein, for which different types of information are displayed in an additional window. All the features listed in Table 1 can be found in the protein ID page and additional information from TAIR, PPDB, Atproteome^7^, SUBA^8^, POGs^9^, and Aramemnon^10^ can be accessed from appropriate links.

## Conclusions and Perspectives

The AT_CHLORO database represents a dedicated resource for getting sub-plastidial localization and functional annotation of Arabidopsis chloroplast proteins, especially for envelope proteins. As revealed by the increasing number of visits and recent publications, information derived from the AT_CHLORO database proved to be of valuable interest for the plant community to ascertain protein sub-plastidial localization, to get insight over metabolic mechanisms, or for data mining purposes. Indeed information regarding the sub-plastidial localization of proteins has proved to be of major interest to confirm the subcellular localization of particular proteins or to investigate biological processes at a larger scale. For instance, in order to decipher mechanistic details of thylakoids biogenesis, analysis, and characterization of mutants can be particularly useful (Adam et al., 2011). The knowledge of the sub-plastidial localization of proteins whose corresponding mutant shows defects in thylakoid network formation, can give insight over the actual role of such proteins. Indeed, as the inner envelope membrane is likely to be the source of internal membrane structures, knowing whether a protein is located in the envelope, or in the thylakoids might help in underlining its role during thylakoid genesis, e.g., with regards to lipid trafficking. In order to determine the subcellular localization of a given protein, Western blotting has been the conventional method for many years. With recent advances in proteomics science, mass spectrometry detection has emerged as an alternative or a complementary approach to Western blots (Mann, 2008). In that context, sub-plastidial information retrieved from AT_CHLORO was also used to ascertain protein localization. For instance, in a recent paper (Karamoko et al., 2011), AT_CHLORO spectral count-based data reinforced immunoblot analyses showing that two FtsZ2 isoforms were associated with the thylakoid membranes. Another example concerns the CJD1 protein that influences fatty acid composition of chloroplast lipids (Ajjawi et al., 2011). GFP fusion experiments suggested that the CJD1 protein was located in the inner envelope of the chloroplast. This information was strengthened by proteomics studies which allowed identification of the CJD1 protein in chloroplast envelope fractions (Ajjawi et al., 2011). Sub-plastidial proteomics data might also be useful to provide a more precise view of sub-organellar compartmentation of biosynthetic pathways, such as isoprenoid (Joyard et al., 2009) or lipid (Joyard et al., 2010) metabolism. In the context of lipid metabolism, sub-plastidial data stored in AT_CHLORO proved to be of strong added value in acknowledging the envelope as a central location for lipid synthesis. For instance, the survey performed by (Joyard et al., 2010) indicated that once the fatty acids are esterified to glycerol-3-phosphate the envelope becomes the key player in glycerolipid biosynthesis, as indicated by the proteomics-based localization of the dedicated enzymes. Thus, sub-plastidial localization found in AT_CHLORO proved to be important and useful with respect to the study of metabolic pathways or of specific proteins.

AT_CHLORO, being targeted to chloroplast proteins, is complementary to more generic plant databases such as PPDB (Sun et al., 2009) or pep2pro (Baerenfaller et al., 2011).

Since the first release of AT_CHLORO we have improved the outputs of the results, especially by providing a graphical overview of sub-plastidial localization of a given set of proteins. The AT_CHLORO database aims at being updated with in-house experiments and curated information related to chloroplast proteins, especially those identified in envelope fractions in order to get the most accurate picture of chloroplast sub-compartmentalization. Indeed we plan to integrate forthcoming experiments that will allow additional identification and quantification data to be produced. Finally, we welcome colleagues from the plant community to provide updated and additional curated information as well as suggestions regarding the different data outputs.

## Conflict of Interest Statement

The authors declare that the research was conducted in the absence of any commercial or financial relationships that could be construed as a potential conflict of interest.

## Footnotes

^1^http://www.grenoble.prabi.fr/at_chloro/

^2^http://www.arabidopsis.org/

^3^http://ppdb.tc.cornell.edu/

^4^http://www.arabidopsis.org/

^5^http://ppdb.tc.cornell.edu/

^6^http://www.cytoscape.org/

^7^http://fgcz-atproteome.unizh.ch/

^8^http://suba.plantenergy.uwa.edu.au/

^9^http://plantrbp.uoregon.edu/

^10^http://aramemnon.uni-koeln.de/
